# Gender specific hippocampal whole genome transcriptome data from mice lacking the Ca_v_2.3 R-type or Ca_v_3.2 T-type voltage-gated calcium channel

**DOI:** 10.1016/j.dib.2017.03.031

**Published:** 2017-03-25

**Authors:** Anna Papazoglou, Christina Henseler, Andreas Lundt, Carola Wormuth, Julien Soos, Karl Broich, Dan Ehninger, Marco Weiergräber

**Affiliations:** a Federal Institute for Drugs and Medical Devices (Bundesinstitut für Arzneimittel und Medizinprodukte, BfArM), Kurt-Georg-Kiesinger-Allee 3, 53175 Bonn, Germany; b German Center for Neurodegenerative Diseases (Deutsches Zentrum für Neurodegenerative Erkrankungen, DZNE), Ludwig-Erhard-Allee 2, 53175 Bonn, Germany

**Keywords:** Calcium channel, Ca_v_2.3, Ca_v_3.2, Gender, Hippocampus, Mouse, R-type, T-type, Transcriptome

## Abstract

Voltage-gated Ca^2+^ channels are of central relevance in mediating numerous intracellular and transcellular processes including excitation-contraction coupling, excitation secretion-coupling, hormone and neurotransmitter release and gene expression. The Ca_v_2.3 R-type Ca^2+^ channel is a high-voltage activated channel which plays a crucial role in neurotransmitter release, long-term potentiation and hormone release. Furthermore, Ca_v_2.3 R-type channels were reported to be involved in ictogenesis, epileptogenesis, fear behavior, sleep, pre-and postsynaptic integration and rhythmicity within the hippocampus. Ca_v_3 T-type Ca^2+^ channels are low-voltage activated and also widely expressed throughout the brain enabling neurons to switch between different firing patterns and to modulate burst activity. Disruption of T-type Ca^2+^ current has been related to sleep disorders, epilepsy, Parkinson׳s disease, depression, schizophrenia and pain. Ca_v_3.2 ablation was further attributed to elevated anxiety and hippocampal alterations resulting in impaired long-term potentiation and memory. Given the importance of Ca_v_2.3 and Ca_v_3.2 voltage-gated Ca^2+^ channels within the CNS, particularly the hippocampus, we collected gender specific microarray transcriptome data of murine hippocampal RNA probes using the Affymetrix Exon Expression Chip Mouse Gene 1.0 ST v1. Information presented here includes transcriptome data from Ca_v_2.3^+/+^, Ca_v_2.3^+/−^, Ca_v_2.3^−/−^, Ca_v_3.2^+/+^, Ca_v_3.2^+/−^ and Ca_v_3.2^−/−^ mice from both genders, the protocol and list of primers used for genotyping animals, the hippocampal RNA isolation procedure and quality controls.

**Specifications Table**

**Table:** 

Subject area	Biology
More specific subject area	Murine hippocampal genomics
Type of data	Transcriptome data
How data was acquired	Affymetrix Exon Expression Chip Mouse Gene 1.0 ST v1
Data format	Raw data
Experimental factors	Hippocampal RNA was extracted from age-matched female and male Ca_v_2.3^+/+^, Ca_v_2.3^+/−^, Ca_v_2.3^−/−^, Ca_v_3.2^+/+^, Ca_v_3.2^+/−^ and Ca_v_3.2^−/−^ mice.
Experimental features	Total RNA from age-matched female and male Ca_v_2.3^+/+^, Ca_v_2.3^+/−^, Ca_v_2.3^−/−^, Ca_v_3.2^+/+^, Ca_v_3.2^+/−^ and Ca_v_3.2^−/−^ mice was hybridized to the Affymetrix Exon Expression Chip Mouse Gene 1.0 ST v1 to unravel transcriptional alterations upon heterozygous or homozygous Cacna1e or Cacna1h allele loss.
Data source location	Bonn, Germany
Data accessibility	Data is available at MENDELEY DATA, doi:10.17632/yp2k3b2577.1 for Ca_v_2.3 transgenic animals and at MENDELEY DATA, doi:10.17632/r6t9mh43s3.1 for Ca_v_3.2 transgenic mice.

**Value of the data**

• Transcriptomes from the hippocampus of age-matched Ca_v_2.3^+/+^, Ca_v_2.3^+/−^, Ca_v_2.3^−/−^, Ca_v_3.2^+/+^, Ca_v_3.2^+/−^ and Ca_v_3.2^−/−^mice.
• Resources for investigation of sex-specific transcriptional differences.
• Provides data basis for analysis of candidate genes that might be important in synaptic integration, learning and memory and ictogenesis upon Ca_v_2.3 or Ca_v_3.2 loss.

## 1 Data

Hippocampus was isolated from Ca_v_2.3^+/+^, Ca_v_2.3^+/−^ and Ca_v_2.3^−/−^, Ca_v_3.2^+/+^, Ca_v_3.2^+/−^ and Ca_v_3.2^−/−^ mice from both genders. Following hippocampal RNA isolation, microarray procedures were carried out to acquire the transcriptome profile of the animals under investigation. The raw reads are accessible at MENDELEY DATA, doi:10.17632/yp2k3b2577.1 for Ca_v_2.3 transgenic animals and at MENDELEY DATA, doi:10.17632/r6t9mh43s3.1 for Ca_v_3.2 transgenic mice.

## 2 Experimental design, materials and methods

### 2.1 Experimental animals

Ca_v_2.3^+/−^ embryos (kindly provided by Richard J. Miller, Department of Neurobiology, Pharmacology and Physiology, The University of Chicago, Chicago, IL, [1]) were re-derived with C57BL/6J mice and maintained with random intra-strain mating obtaining all genotypes [2]. Exons 4–8 encoding the pore-lining domain neighboring transmembrane domains of Cacna1e were replaced with a neo-URA3 cassette via homologous recombination (Mouse Genome Informatics; MGI Ref. ID J: 66144). For hippocampal exstirpation and subsequent transcriptome analysis, Ca_v_2.3^+/+^ controls, heterozygous Ca_v_2.3^+/−^ and homozygous null mutant Ca_v_2.3^−/−^ mice were used from both age-matched genders with the following characteristics: Males: Ca_v_2.3^+/+^: *n* = 3 (♂) sample # 1–3, 19.00±1.57 wks; Ca_v_2.3^+/−^: *n* = 3 (♂) sample # 4–6, 18.57±1.41 wks; Ca_v_2.3^−/−^: *n* = 3 (♂) sample # 7–9, 20.52±0.24 wks. Females: Ca_v_2.3^+/+^: *n* = 3 (♀) sample # 10–12, 20.62±3.51 wks; Ca_v_2.3^+/−^: *n* = 3 (♀) sample # 13–15, 20.29±0.00 wks; Ca_v_2.3^−/−^: *n* = 3 (♀) sample # 16–18, 19.86±0.30 wks. Note that sample numbers given here correlate with those of the transcriptome data set (MENDELEY DATA, doi:10.17632/yp2k3b2577.1).

Ca_v_3.2 transgenic mice [3] from Mutant Mouse Resource and Research Centers (MMRRC: 009979-MU; strain name: B6.129-*Cacna1h*^*tm1Kcam*^/Mmmh) were maintained in the C57Bl/6J background. For hippocampal exstirpation and subsequent transcriptome analysis, Ca_v_3.2^+/+^ controls, heterozygous Ca_v_3.2^+/−^ and homozygous null mutant Ca_v_3.2^−/−^ mice were used from both age-matched genders with the following characteristics: Males: Ca_v_3.2^+/+^: *n* = 3 (♂) sample # 19–21, 19.76±0.05 wks; Ca_v_3.2^+/−^: *n* = 3 (♂) sample # 22–24, 21.00±0.00 wks; Ca_v_3.2^−/−^: *n* = 3 (♂) sample # 25–27, 20.90±0.38 wks. Females: Ca_v_3.2^+/+^: *n* = 3 (♀) sample # 28–30, 20.52±0.38 wks; Ca_v_3.2^+/−^: *n* = 3 (♀) sample # 31–33, 21.38±0.05 wks; Ca_v_3.2^−/−^: *n* = 3 (♀) sample # 34–36, 20.76±0.27 wks. Note that sample numbers given here correlate with those of the transcriptome data set (MENDELEY DATA, doi:10.17632/r6t9mh43s3.1).

All experimental animals were housed in groups of 3–4 in clear Macrolon cages type II with ad libitum access to drinking water and standard food pellets. Using ventilated cabinets (Type Uniprotect, Bioscape), mice were maintained at a temperature of 21±2 °C, 50%–60% relative humidity, and on a conventional 12 h light/dark cycle with the light cycle starting at 05:00 AM. All animals were strictly adapted to this circadian pattern preceding subsequent hippocampal exstirpation. Animal procedures were performed according to the Guidelines of the German Council on Animal Care and all protocols were approved by the Local Institutional and National Committee on Animal Care (Landesamt für Natur, Umwelt und Verbraucherschutz, LANUV, Germany). The authors further certify that all animal experimentation was carried out in accordance with the European Communities Council Directive of November 24, 1986 (86/609/EEC).

### 2.2 Genotyping

#### 2.2.1 DNA Preparation from tail biopsies

For genotyping, DNA was isolated from tail biopsy of each experimental animal. Tissue was incubated over night at 55 °C in 100 µl lysis buffer (Tris pH 9, 10 mM; KCl, 50 mM; Triton X-100, 0.1%; proteinase K, 1 mg/ml) using an orbital shaker. Subsequently, lysis was stopped by heating the samples to 95 °C for 15 min. Following brief centrifugation (30 s, 20,000x*g*), isolated genomic DNA was used for PCR [2].

#### 2.2.2 Polymerase Chain Reaction (PCR)

##### 2.2.2.1 Ca_v_2.3 transgenic mice

Isolated genomic DNA of each experimental animal was added to a PCR Mastermix containing primers (WT forward: 5′-GGC TGC TCT CCC AGT ATA CT-3′; WT reverse: 5′-CAG GAA GCA TCA CTG CTT AG-3′; KO forward: 5′-ATT GCA GTG AGC CAA GAT TGT GCC-3′), H_2_O (PCR grade) and RedTaq Ready Mix according to the manufacturer׳s instructions (Sigma Aldrich, Germany) (Table 1). The mix was vortexed thoroughly followed by a short centrifugation step (10 s, 2000x*g*) using a mini centrifuge (Carl Roth, Germany). For PCR, a BioRad thermal cycler (Type C1000) was used. Following a 3 min pre-incubation step at 94 °C the following program was applied for 34 cycles:

– 94 °C, 30 s denaturation
– 59 °C, 30 s annealing
– 72 °C, 1 min extension

**Fig. 1:**
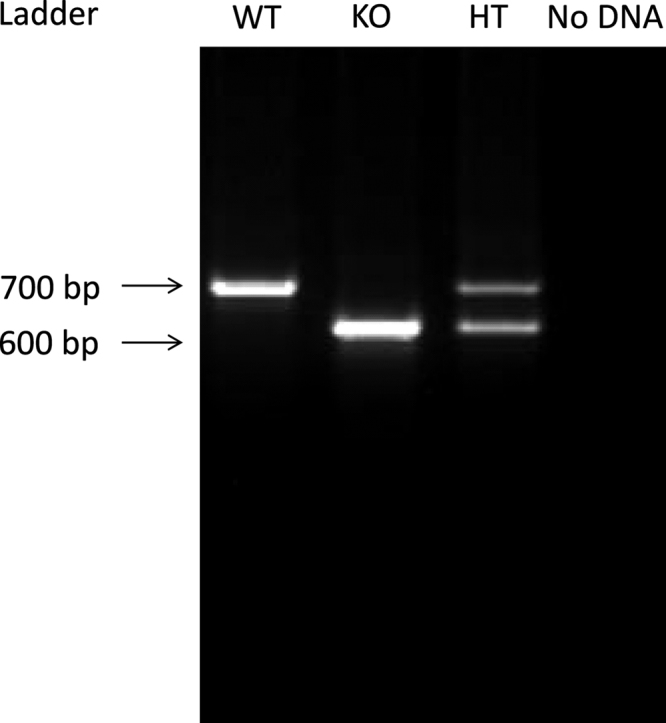
Genotyping of Ca_v_2.3^+/+^, Ca_v_2.3^+/−^ and Ca_v_2.3^−/−^ mice. Amplification of characteristic DNA fragments allows for characterization of individual genotypes. Negative controls (e.g. with no genomic DNA) do not show amplification.

The 34 cycles were followed by a 72 °C step for 10 min and subsequent cooling to 4 °C. All PCR experiments were validated using positive and negative controls. Amplified PCR products were loaded on a 1.5% agarose gel containing agarose in 0.5 X TBE buffer (pH 8.0) and 0.3 mg/l ethidium bromide placed in a gel electrophoresis chamber filled with 0.5 X TBE. PCR product visualization and genotype specification was done using DIANA Imaging software (Raytest, Germany) [2] (Fig. 1).

##### 2.2.2.2 Ca_v_3.2 transgenic mice

Genomic DNA of each mouse was added to a PCR Mastermix containing primers (WT forward: 5′-ATT CAA GGG CTT CCA CAG GGT A-3′; WT reverse: 5′-CAT CTC AGG GCC TCT GGA CCA C-3′; KO reverse: 5′-GCT AAA GCG CAT GCT CCA GAC TG-3′), H_2_O (PCR grade) and RedTaq Ready Mix (Sigma Aldrich; Germany) (Table 1). The mix was vortexed thoroughly followed by a short centrifugation step (10 s, 2000x*g*) using a mini centrifuge (Carl Roth, Germany). For PCR a BioRad thermal cycler (Type C1000) was used. Following an initial 3 min pre-incubation step at 94 °C the following program was applied for 34 cycles:

– 94 °C, 30 s denaturation
– 61 °C, 30 s annealing
– 72 °C, 1 min extension

**Fig. 2:**
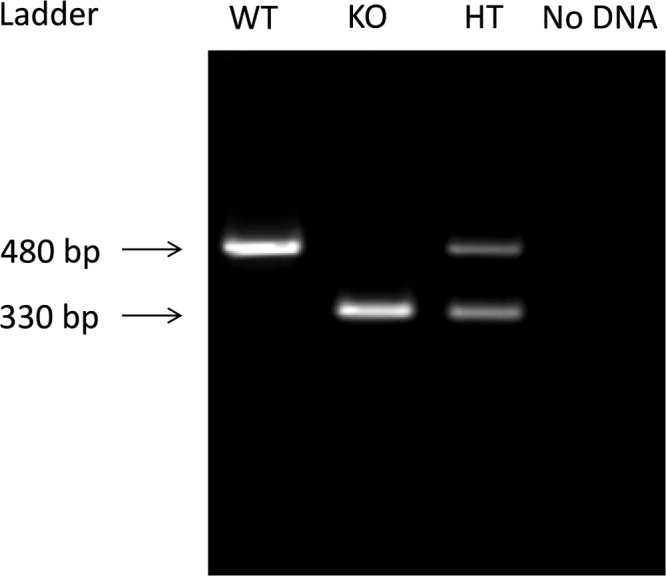
Genotyping of Ca_v_3.2^+/+^, Ca_v_3.2^+/−^ and Ca_v_3.2^−/−^ mice. Amplification of characteristic DNA fragments allows for characterization of individual genotypes. Negative controls (e.g. with no genomic DNA) do not show amplification.

The 34 cycles were followed by a 72 °C step for 10 min and subsequent cooling to 4 °C. To validate the results of each PCR positive and negative controls were performed. PCR product visualization (as described above) and genotype determination was performed using DIANA Imaging software (Raytest, Germany) (Fig. 2).

### 2.3 Hippocampus preparation and tissue storage

Experimental animals used for the experiment were deeply anaesthetized using ketamine (100 mg/kg) / xylazine (10 mg/kg) i.p. Following decapitation the brain was quickly removed and placed in a petri dish with 0.9% NaCl on ice. Both hemispheres were separated using a scalpel and the hippocampus was bluntly dissected using a forceps and brush. Immediately after dissection the hippocampus was placed in a 2 ml reaction tube and snap frozen in liquid nitrogen. Hippocampal tissue samples were stored at −80 °C until RNA preparation.

### 2.4 Hippocampal RNA isolation

RNA preparation was carried out using the Lipid Tissue Mini Kit (Qiagen) following the manufacturer׳s instructions. Hippocampal tissue samples were lysed and homogenized using a handheld rotor-stator homogenizer (Tissue Ruptor, Qiagen) with disposable probes. All centrifugation steps were carried out at 10,000x*g* and RNA was finally eluted in 50 µl RNase free H_2_O. After preparation the quantity and quality of the eluted RNA was checked using Nanodrop (Thermo Scientific). Prior to RNA hybridization to the Microarray Chips the RNA quality was tested again using the Bioanaylzer 2100 (Agilent Technology, see below).

### 2.5 Affymetrix microarray procedures

Microarray experiments were performed using the Exon Expression Chip Mouse Gene 1.0 ST v1 (Affymetrix). These exon arrays include 6.553.600 probes with a coverage density of 4 probes for each exon of all known and predicted genes of the mouse genome. RNA integrity was determined with Agilent׳s Bioanalyzer with samples RNA integrity numbers (RIN) ranging from 8.3 to 9.2. Arrays were washed and stained according to the manufacturer׳s recommendations (Affymetrix). Single-stranded cDNA was generated from the amplified cRNA with the WT cDNA Synthesis Kit (Affymetrix), purified, fragmented and labeled with WT Terminal Labeling Kit (Affymetrix). Following hybridization to the arrays, scanning was carried out in a GeneChip 3000 7 G scanner (Affymetrix). Array data are available at MENDELEY DATA, doi:10.17632/yp2k3b2577.1 for Ca_v_2.3 transgenic animals and at MENDELEY DATA, doi:10.17632/r6t9mh43s3.1 for Ca_v_3.2 transgenic mice.

## Funding

This work was supported by the (BfArM 9_00039).

## Figures and Tables

**Table 1:** Composition of PCR reaction using PCR Mastermix and genomic DNA.

**Reagent**	**Volume per reaction (µl)**
H_2_O	9.3
Forward primer WT - 50 ng/µl	0.3
Forward primer KO - 50 ng/µl	0.3
Reverse primer WT - 50 ng/µl	0.6
Red Taq Ready Mix	12.5
Genomic DNA	2
**Total Volume**	**25**
